# Transcriptome analysis revealed that AcWRKY75 transcription factor reduced the resistance of kiwifruit to *Pseudomonas syringae* pv. *actinidiae*

**DOI:** 10.3389/fpls.2024.1488572

**Published:** 2024-10-24

**Authors:** Lixia Ye, Minmin Luo, Yafang Wang, Mengqi Yu, Zhi Wang, Fuxi Bai, Xuan Luo, Li Li, Qiong Huang, Jue Peng, Qi Chen, Qinghong Chen, Lei Gao, Lei Zhang

**Affiliations:** ^1^ Hubei Key Laboratory of Germplasm Innovation and Utilization of Fruit Trees, Institute of Fruit and Tea, Hubei Academy of Agricultural Science, Wuhan, China; ^2^ College of Horticulture and Gardening, Yangtze University, Jingzhou, China; ^3^ Key Laboratory of Plant Germplasm Enhancement and Specialty Agriculture, Wuhan Botanical Garden, Chinese Academy of Sciences (CAS), Wuhan, China; ^4^ Technology Research and Development Department, Chibi Shenshan Xingnong Technology Co. LTD, Chibi, China

**Keywords:** kiwifruit, *Pseudomonas syringae* pv. *actinidiae*, WRKY75, transcriptome, transcription factor, disease resistance

## Abstract

The kiwifruit canker disease caused by *Pseudomonas syringae* pv. *actinidiae* (Psa) seriously threatens the development of kiwifruit industry. So far, only a limited number of Psa-resistant kiwifruit varieties have been identified, and the underlying molecular mechanisms are still largely unknown. In this study, we evaluated the Psa resistance of six hybrid populations and screened a resistant segregation population R_1_F_2_. Then, transcriptome analysis on the Psa extremely high-resistant (HR) and extremely high-susceptible (HS) plants of the R_1_F_2_ population was performed. KEGG enrichment analysis revealed that differentially expressed genes (DEGs) were significantly enriched in plant hormone signal transduction pathways, including auxin, abscisic acid, zeatin, jasmonic acid and salicylic acid. Furthermore, several transcription factors (TFs), especially WRKY TFs, were identified among the DEGs. The qRT-PCR showed that *AcWRKY75* was highly expressed in the HS plants. Additionally, *AcWRKY75* was significantly induced in the HS cultivar ‘Hongyang’ after Psa inoculation. Sequence amplification analysis showed that there was polymorphism in the DNA sequence of *AcWRKY75* gene, but no HR or HS-specific differences were observed. Subcellular localization and transcriptional activity analysis confirmed that AcWRKY75 functions as a nucleus-located transcriptional activator. Transient overexpression of *AcWRKY75* in kiwifruit leaves reduced the resistance to Psa, while silencing *AcWRKY75* by virus-induced gene silencing (VIGS) slightly enhanced the resistance to Psa. Furthermore, AcWRKY75 exhibited a weak interaction with the promoter of the ABA-related DEG *AcBet V1* (Acc27163). Our findings elucidated that AcWRKY75 may negatively regulate the Psa resistance of kiwifruit through the hormone signaling pathway, which laid a foundation for the analysis of the disease resistance mechanism of kiwifruit canker.

## 1 Introduction

Kiwifruit (*Actinidiae chinesis* Planch.) is known as the king of fruits because of its rich nutrients such as vitamin C, dietary fiber, and minerals. It has a short cultivation history, but the industry is developing rapidly and highly concentrated. With the highly centralized development, various infectious diseases have begun to spread, among which kiwifruit canker is the most threatening (Vanneste et al., 2014). Kiwifruit canker is a devastating bacterial disease caused by *Pseudomonas syringae* pv. *actinidiae* (Psa), which mainly harms kiwifruit’s trunk, branches and leaves (Yu et al., 2016). In the early stage of Psa infection, milky white secretions are generated at the infection sites of trunks, and later transformed into rust red. Subsequently, due to the abnormal transport of nutrients and water, kiwifruit trees became weak and even died (Renzi et al., 2012). Kiwifruit canker disease occurs in almost all major producing areas of the world (Vanneste et al., 2011; Bastas and Karakaya, 2012; Renzi et al., 2012; Vanneste et al., 2014; Yang et al., 2015). It has brought huge economic losses to fruit farmers and seriously threatened the development of the kiwifruit industry (Deng et al., 2023). So far, kiwifruit canker disease-resistant materials are still lacking, and the disease-resistance mechanism remains to be further understood (Lu et al., 2024; Zhao et al., 2024).

In recent years, with the development of omics technology, some kiwifruit canker-resistance genes have been identified. Transcriptome analysis of the Psa-resistant variety ‘Maohua’ and the susceptible variety ‘Hongyang’ revealed that the immune-related gene *MAP2* was significantly up-regulated in ‘Maohua’, whereas *NPR1* and *TGA* were significantly up-regulated in ‘Hongyang’ (Qin et al., 2022). Comparative transcriptome analysis of the high-resistant variety ‘Huate’ and the high-susceptible variety ‘Hongyang’ at different time points after Psa infection has been conducted, and four types of disease-resistant related genes have been characterized (Song et al., 2019). Moreover, proteomic analysis of kiwifruit showed that membrane-associated proteins BamA, OmpA and OmpF may be related to Psa infection (Petriccione et al., 2013). Genome resequencing was performed on the hybrid F_1_ population of tetraploid *A. chinensis* Psa high-susceptible female parent ‘P1’ and high-resistant male parent ‘P2’, and a Psa resistance-related QTL was mapped (Tahir et al., 2019, Tahir et al., 2020). Recent studies have demonstrated that transcription factors (TFs) play a significant role in the disease resistance of kiwifruit against Psa. For instance, three TGACG-binding TFs, *AcTGA01*, *AcTGA06*, and *AcTGA07*, have been confirmed to be involved in the regulation of kiwifruit resistance to Psa (Liu et al., 2022). Similarly, three core TFs, *AcC3H1*, *AcZML1* and *AcREM14*, were induced by Psa and exhibited a high expression trend in four resistant varieties (Zhao et al., 2024).

WRKY TF represents one of the largest TF families and plays a crucial role throughout the life cycle of plants. The first WRKY TF was cloned from sweet potato and named SPF1 (de Pater et al., 1996). Since then, WRKY family genes have been identified in an increasing number of plants, such as Arabidopsis (de Pater et al., 1996), rice (Ramamoorthy et al., 2008), poplar (Jiang et al., 2014), peach (Chen et al., 2016), kiwifruit (Jing and Liu, 2018), and citrus (Dai et al., 2023). WRKY TFs contain one or two WRKY domains, along with a C-terminal zinc finger motif. The amino acid sequence WRKYGQK of the WRKY domain is relatively conserved (Zhang and Wang, 2005). WRKY TF can be classified into three groups according to the quantity of WRKY domains and the characteristics of zinc-finger motifs. Typically, group I contains two WRKY domains along with a C2H2 motif; group II includes a single WRKY domain paired with a C2H2 motif; while group III comprises one WRKY domain and a C2HC motif (Yin et al., 2024). WRKY TFs play a crucial role in orchestrating the early defense responses associated with disease resistance in plants. For instance, in *Arabidopsis*, *AtWRKY3* and *AtWRKY4* overexpression lines are sensitive to the infection of *Pseudomonas syringae* and *Botrytis cinerea* (Lai et al., 2008). *AtWRKY48* has been identified as a negative regulator in the basic resistance to *Pseudomonas syringae* (Xing et al., 2008). In rice, overexpression of *OsWRKY13*, *OsWRKY53* and *OsWRKY71* can enhance the resistance to bacterial blight and blast (Liu et al., 2007; Qiu et al., 2007; Chujo et al., 2009), while overexpression of *WRKY62* and *WRKY76* increased the susceptibility of transgenic plants (Liu et al., 2016). Similarly, overexpression of *OsWRKY45* enhanced the resistance of transgenic plants to *Pseudomonas syringae* pv. *tomato* (Pst) (Qiu and Yu, 2009). In tobacco, *NbWRKY22/25* is induced by Pst and is a positive regulator of ETI response (Ramos et al., 2021). Overexpression of *CaWRKY40* can enhance the resistance of tobacco to bacterial wilt, While silencing the *CaWRKY40* gene enhances the susceptibility of pepper to bacterial wilt (Dang et al., 2013; Yang et al., 2022). *SlWRKY8* can enhance the resistance of tomato to Pst (Gao et al., 2020). In kiwifruit, transient overexpression of *AcWRKY94* in the leaves of ‘Hongyang’ enhanced the resistance of kiwifruit to Psa (Lu et al., 2024).

*WRKY75* gene is involved in a variety of biological processes, such as seed germination (Zhang et al., 2022a), root hair development (Devaiah et al., 2007; Rishmawi et al., 2014), flowering (Zhang et al., 2018), leaf senescence (Guo et al., 2017; Zhang et al., 2022a), biotic stresses (Encinas-Villarejo et al., 2009; Liu et al., 2018b; Chen et al., 2021; Yang et al., 2024) and abiotic stresses (Devaiah and Raghothama, 2007; Ping et al., 2024). In this study, the expression of the kiwifruit *WRKY75* gene was analyzed in Psa high-resistant plants and high-susceptible plants. The function of the *AcWRKY75* gene was verified by transient transformation, and the possible regulatory pathways in which AcWRKY75 may be involved were preliminarily revealed. Our study revealed that AcWRKY75 acts as a negative regulator to participate in the regulation of kiwifruit canker disease resistance, which laid a foundation for uncovering the disease resistance mechanism of kiwifruit canker.

## 2 Materials and methods

### 2.1 Plant materials

Kiwifruit is a functional dioecious plant. Male plants only form male flowers and female plants only form female flowers. The pistil on the male flower is not developed, and the pollen on the female flower is completely aborted (Caporali et al., 2019). The female plants used in this study were Psa high-resistant variety ‘Jinkui’, and the male plants were 3 materials screened by our team with high resistance, medium resistance and high sensitivity to Psa, respectively. For artificial hybridization, the pollen of male plants was collected first, and then the female plants were artificially pollinated and bagged. Once the fruit had ripened, the seeds were collected, treated, and then sown to obtain the hybrid. The six hybrid populations of this study were constructed in 2018, with a total of 4351 plants. JA, JB and JM were three BC_1_ hybrid populations, and A_1_F_2_, D_1_F_2_ and R_1_F_2_ were three F_2_ populations. The population materials were planted in the kiwifruit base of Chibi Shenshan Xingnong Technology Co. LTD. and grew well.

### 2.2 Psa resistance investigation of hybrid population plants in the field

The field resistance of six populations was investigated in April 2022 according to the reported method (Wang et al., 2019) with a few modifications. The disease resistance grade was simplified from five (Highly Resistant (HR), Resistant (R), Moderate (M), Susceptible (S) and Highly Susceptible (HS)) to three (HR, M and HS). In brief, plants with no visible lesions on the leaves are HR plants; plants with some lesions on the leaves are M plants, and plants with many lesions on the leaves and almost all leaves have lesions are HS plants.

### 2.3 Identification of Psa resistance of isolated branches

The pathogen Psa (C48) was cultured in LB liquid medium at 25°C with shaking at 200 rpm for 24 hours. Then the bacteria were collected by centrifugation, and the concentration of Psa bacteria was diluted to 1×10^8^ cfu/mL with sterile water for infection. After the kiwifruit leaves fall in winter, six healthy kiwifruit branches with a diameter of about 0.8 cm were collected from each plant. According to the reported method (Wang et al., 2019, Wang et al., 2023), the branches were washed with running water and then were cut into 10 cm sections. The ends of the branches were sealed with paraffin to reduce the loss of water. Next, the branches were disinfected with 75% alcohol in the ultra-clean bench. After the alcohol volatilizes, punching was performed with a puncher at a distance of about 1.5 cm from the end of the branch to remove the epidermis. The 10 μL diluted Psa solution was inoculated at the wound for infection, and sterile water was inoculated as a control. Each plant was infected with six branches and repeated three times. Once the bacterial liquid had been completely absorbed in the wound, the branches were placed in a tray covered with soaked filter paper. The tray was placed in an artificial climate chamber and cultured at 20°C, 12 h light/12 h dark, relative humidity of 80%. The incision symptoms were observed every three days, and the lesion length was measured at 40 days after inoculation.

### 2.4 Transcriptome sequencing and analysis

In the R_1_F_2_ population, nine extremely HR plants and nine extremely HS plants to Psa in field investigation and *in vitro* branch identification were screened for transcriptome sequencing. Leaf samples of new shoots were taken, frozen in liquid nitrogen and stored at -80°C for later use. Total RNA was extracted using the Total RNA Extraction Kit (Aidlab, Beijing, China) according to the manufacturer’s instructions. The purity, concentration and integrity of RNA samples were detected by NanoDrop, Qubit 2.0 and Agilent 2100. Qualified RNA of each three extremely high-resistant plants was mixed into one sample, and finally a total of three high-resistant samples were obtained, namely HR1, HR2, and HR3. Similarly, the RNA of each three extremely high-susceptible plants was mixed into one sample to obtain HS1, HS2 and HS3. Then, the RNA was sequenced by the Illumina platform in BioMac Biotechnology Co., Ltd. The sequencing reads were assembled and mapped to the kiwifruit reference genome (http://kiwifruitgenome.org) using StringTie and HISAT software (Rao, 2024; Thakur, 2024). Applying DESeq2, differentially expressed genes (DEGs) were annotated by calculating fragments per kilo-base of transcripts (FPKM) with fold change ≥ 2 and FDR < 0.01, and KEGG enrichment analysis was performed (Liu et al., 2021). TFs in DEGs were screened and classified according to gene annotation. Heat maps of DEGs were visualized by the HeatMap Illustrator tool of TBtools (Chen et al., 2023).

### 2.5 Quantitative RT–PCR analysis

The total RNA of three HR and three HS plants from the R_1_F_2_ populations was extracted using the Total RNA Extraction Kit (Aidlab, Beijing, China) according to the manufacturer’s instructions. The quality and concentration of the RNA were detected by agarose gel electrophoresis and Nanodrop spectrophotometer. Reverse transcription was performed using TRUEscript RT MasterMix (OneStep gDNA Removal) (Aidlab, Beijing, China). The cDNA was diluted to 300 ng/μL as a template for qRT-PCR. Primers used in this study are listed in **Supplementary Table S6**. The qRT-PCR was performed according to previous reports (Ye et al., 2022). In brief, the total volume of the qRT-PCR mixtures was 10 µL, which included 5 µL of SYBR qPCR Master Mix (Takara, Japan), 0.2 µL of each primer (10 µM), 0.4 µL of cDNA, and 4.2 µL of RNase-free water. The ABI 7500 Sequence Detection System was employed to conduct the reactions under the following thermocycling parameters: an initial enzyme activation step at 95°C for 5 minutes, followed by 40 cycles comprising 10 seconds at 95°C and 20 seconds at 60°C. After the reaction was completed, a melting curve analysis was performed. The 2^−ΔΔCT^ method was used to calculate the relative gene expression of genes. The AcActin gene (Acc05529.1) was used for the normalization of qRT-PCR data.

### 2.6 DNA and protein sequence alignment analysis of *AcWRKY75* gene

AcWRKY75 protein sequence was used for protein blast on NCBI, and the top 50 protein sequences (**Supplementary Table S7**) with high homology to AcWRKY75 protein were downloaded. These sequences were aligned using the ClustalW multiple method in BioEdit software (Sofi et al., 2022), and then a phylogenetic tree was constructed in MEGA 11 using the maximum likelihood method (Tamura et al., 2021). Twelve WRKY75 proteins with the highest similarity were selected for multiple sequence alignment to analyze the variation of conserved domains. The WRKY domain has a conserved WRKYGQK sequence, and the C2H2 domain comprises CX_4-5_-C-X_22-23_-H-X_1_-H (Yin et al., 2024). According to these characteristics, the location of the domains in the WRKY75 protein was marked. To analyze the variation of the *AcWRKY75* gene in HR and HS plants of the R_1_F_2_ population, the new shoot leaves of three HR plants and three HS plants were sampled, and genomic DNA was extracted following the manual of a modified CTAB Plant DNA Kit (Aidlab, Beijing, China). Then, the DNA was diluted to 300 ng/μL as the amplification template, and the *AcWRKY75* gene was amplified by PhantaMax Super-Fidelity DNA Polymerase (Vazyme, Nanjing, China). The target fragment was cloned into the pTOPO-Blunt vector using a Cloning Kit (Aidlab, Beijing, China) and transferred into *E.coli* DH5α. Single clones containing the target fragments were sent to the company for sequencing. Finally, the sequences were subject to multiple sequence alignment using BioEdit software (Sofi et al., 2022).

### 2.7 Subcellular localization analysis

Subcellular localization analysis was performed as previously described (Ye et al., 2021). Briefly, the coding sequences (CDS) of the *AcWRKY75* gene was amplified, and the part without termination codon was cloned into the pBI121-GFP vector. Then the recombinant vector was transferred into *A. tumefaciens* strain EHA105 cells. Next, the *A. tumefaciens* cells were subject to co-infiltration to *N. benthamiana* leaves. Afterward, the *N. benthamiana* was cultured in dark condition and light condition for one day, respectively. Finally, the leaves were collected for fluorescence signal detection under a laser confocal microscope (TCS-SP8, Leica, Germany). A red fluorescent protein (RFP) marker was used to indicate the nucleus location by cotransfecting, and the 35S-GFP vector, which emits fluorescence in both the cell membrane and nucleus, was used as a positive control.

### 2.8 Transcriptional activity analysis

To assess the transcriptional activation activity of AcWRKY75, the full-length CDS of the *AcWRKY75* gene was amplified and inserted into the pGBKT7 vector, resulting in a fusion with the GAL4-BD domain. The empty pGBKT7 vector and the recombinant plasmid BD-AcWRKY75 were transformed into the yeast AH109 strain and cultured on SD/-Trp medium at 30°C for three days. Following this, the optical density at 600 nm (OD_600_) of the positive clone was diluted to 10^0^,10^-1^,10^-2^ using 0.9% NaCl solution, and 10 µL of the bacterial solution was spotted onto SD/-Trp, SD/-Trp-His-Ade, and SD/-Trp-His-Ade supplemented with X-α-gal media. After three days of incubation at 30°C, the transactivation activity of the transformants was evaluated based on their growth patterns. Positive clones exhibited growth on all media and turned blue in the SD/-Trp-His-Ade medium containing X-α-gal, while the control grew only on SD/-Trp, indicating that the positive clones possessed transcriptional activation activity.

### 2.9 Transient overexpression and silencing of *AcWRKY75* in kiwifruit leaves

The vectors PBI121 and TRV1/2 were used for overexpression and virus-induced gene silencing (VIGS), respectively. The recombinant plasmids were separately transferred into *A. tumefaciens* strain EHA105. Transient overexpression in kiwifruit leaves was performed using a previously described method (Lu et al., 2024). Briefly, the OD_600_ of *A. tumefaciens* infection solution was adjusted to 0.8 with MES buffer (10 mM MgCl_2_, 10 mM MES, 150 mM AS, pH 5.6), and then injected into the leaves of kiwifruit variety ‘Jinyi’. Each infection solution was injected into the same area of different regions. The infected plants were cultured in the dark for one day and then cultured at 25°C with a 16h light/8h dark photoperiod for another day. Then, the leaves were collected and placed in -80°C for the *AcWRKY75* gene expression level analysis. Two days post-injection, the Psa solution was diluted with sterile water containing 10 m M Mg Cl2 to OD600 = 0.5 and then added with 0.03% Silwet L-77. The infection site was then brushed twice with this bacterial solution. Afterward, the plants were promptly positioned beneath a plastic dome at 20°C, maintaining a photoperiod of 16 hours of light followed by 8 hours of darkness. Two weeks post-injection, images of the infected leaves were collected and the disease index was calculated using ImageJ (V1.8.0). The number of Psa in the infected leaves was quantified according to a reported described method (Zhao et al., 2024). Briefly, 0.1 g leaves were transferred to a test tube containing 2.0 ml of 0.9% sodium chloride and gently ground with quartz sand. Subsequently, the suspension was subjected to a series of dilutions (10^0^, 10^−1^, 10^−2^ and 10^−3^), and 100 µL of each sample was taken and cultured in LB medium at 28°C for 72 h. Once the colony appeared, the number of bacteria was counted. Assays were repeated at least three times for each infected sample.

### 2.10 Yeast one-hybrid assay

The 2 kb promoter fragments of 14 ABA-related Bet v 1 family genes were isolated from the *A. chinensis* genome (http://kiwifruitgenome.org) using TBtools (Chen et al., 2023), and the W-box was found. The *AcBet V1* (Acc27163) gene promoter contains four W-box (GGTCAA) elements. The W-box (-230 bp to-236 bp) closest to the start codon was ligated into the pAbAi vector, and the AcWRKY75 coding region was ligated into the PGADT7 vector. The bait vector pAcBet V1-AbAi was transformed into the yeast Y1H-Gold strain and tested self-activation with SD/-Ura medium under different concentrations of AbA (0, 100, 300, 500, 800 ng/mL). Yeast transformation was performed according to the manual of the Y1HGold Yeast One-hybrid System kit (Coolaber, Beijing, China). The pGADT7-p53+p53-AbAi was a positive control, and the pGADT7+pAcBet V1-AbAi was a negative control.

## 3 Results

### 3.1 Screening Psa resistant and susceptible plants in hybrid progeny

**Figure 1:**
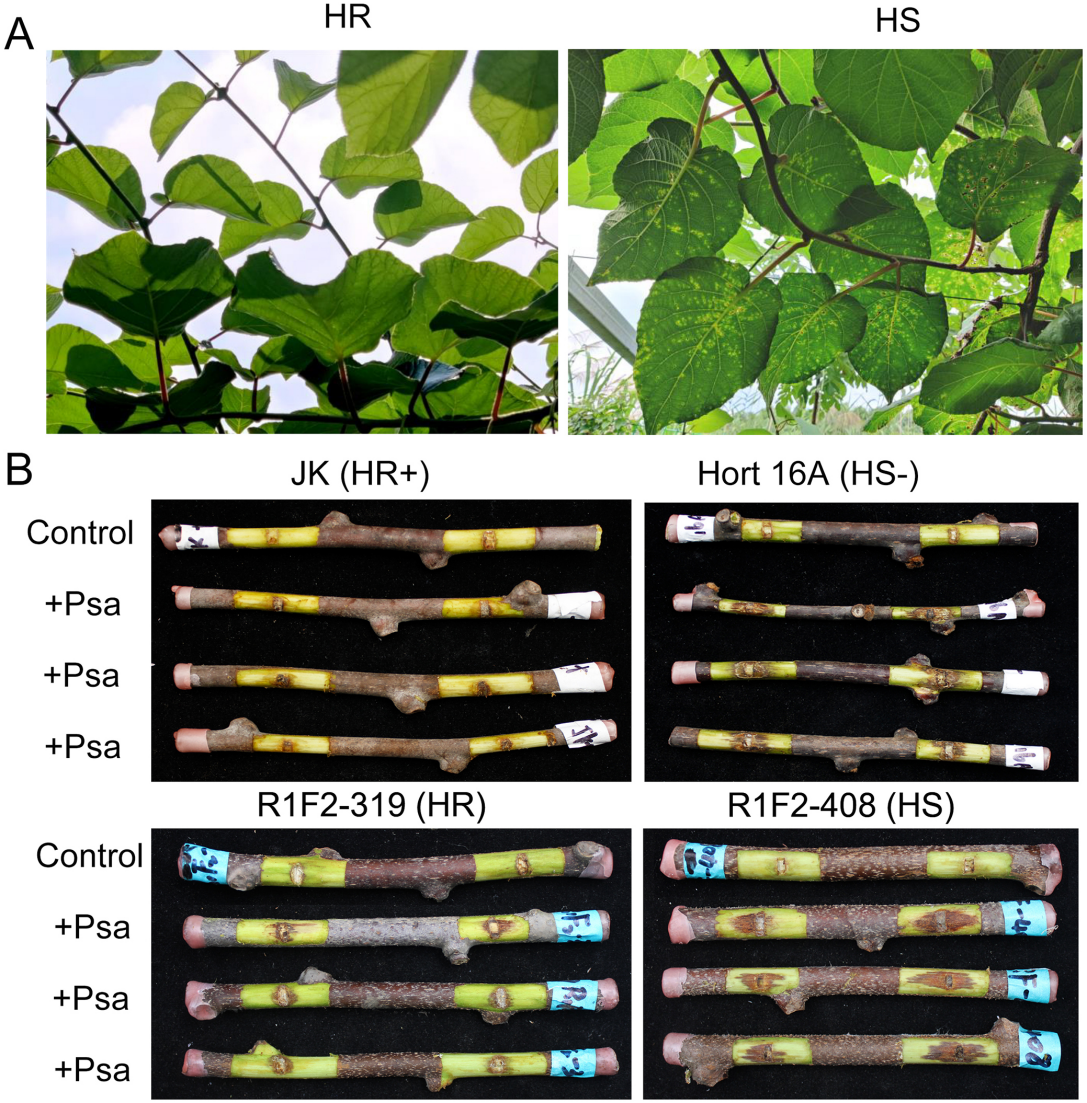
Phenotypes of Psa highly resistant and highly susceptible plants in the R_1_F_2_ population in the field and *in vitro* identification. **(A)** Phenotypes of high-resistant (HR) and high-susceptible (HS) plants in the field. Plants with no visible lesions on the leaves are HR plants, and plants with lesions on almost all leaves are HS plants. **(B)** The lesions of isolated branches inoculated with Psa. ‘JK’ was a HR positive control; ‘Hort 16A’ was a HS negative control. Control indicates blank control treated with water. +Psa indicates Psa pathogen inoculation. R1F2-319 is identified as a HR plant, and R1F2-408 is identified as a HS plant.

To obtain different resistance materials of Psa, we constructed six hybrid populations with Psa high resistance variety ‘Jinkui’ as the female parent and varieties with different Psa resistance levels as the male parents. To screen out the Psa resistance segregating populations, the resistance of lignified branches in each population was identified. The results showed that the resistance segregation of JA, JB, A_1_F_2_ and D_1_F_2_ populations was not obvious, and most plants were HR. While JM and R_1_F_2_ populations were separated and R_1_F_2_ population had the most obvious plant resistance separation (**Supplementary Figure S1**). The disease resistance of the six populations to Psa in the field was further investigated, and the disease resistance was divided into high resistance (HR), medium resistance (M) and high sensitivity (HS) according to the disease spots on the leaves (**Figure 1A**). The statistical results indicated a clear separation in disease resistance within the R_1_F_2_ population (**Supplementary Figure S2**), which aligned with the identification results of branch resistance. These findings suggest that the R_1_F_2_ population had the most obvious Psa resistance segregation among the six populations, making it the most suitable population for mapping canker disease resistance genes.

To further screen the individual plants with extremely HR and HS to Psa from the R_1_F_2_ population, the individual plants showing HR and HS to Psa in the field were re-evaluated in isolated branches (**Figure 1B**). The HR variety ‘Jinkui’ was used as the HR-positive control, and the HS variety ‘Hort 16A’ was used as the HS-negative control. Based on the lesion length, the plants of the R_1_F_2_ population were classified into HR, MR and HS. For example, R_1_F_2_-319 is identified as an extremely HR plant, and R_1_F_2_-408 is identified as an extremely HS plant (**Figure 1B**). Nine plants exhibiting extremely HR were selected to construct HR pools, and nine plants exhibiting extremely HS were also chosen to construct HS pools for transcriptome sequencing.

### 3.2 Transcriptome sequencing of Psa resistant and susceptible plants

**Figure 2:**
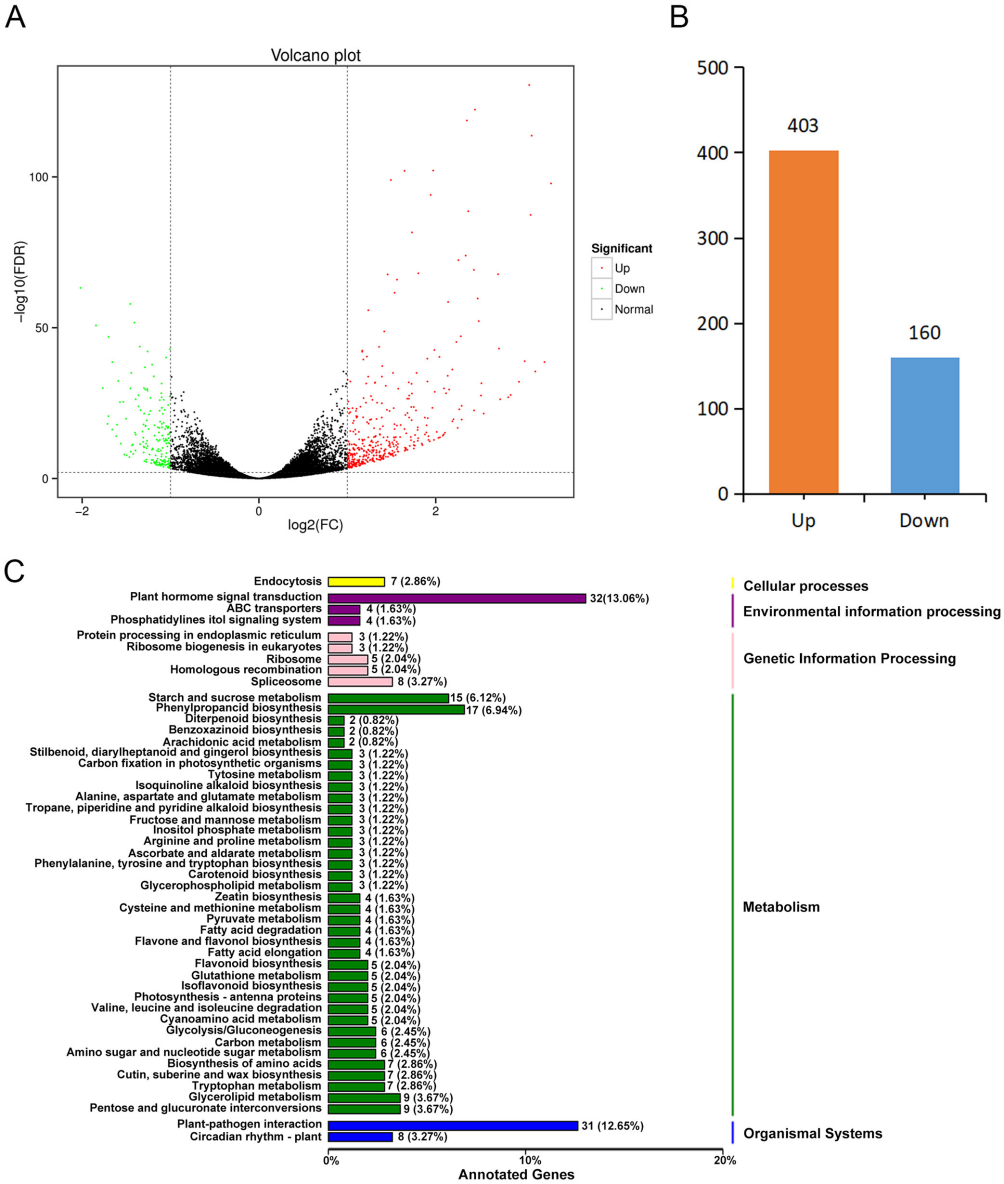
Transcriptome analysis of Psa highly resistant and highly susceptible plants in the R_1_F_2_ population. **(A)** Volcano map of differentially expressed genes. **(B)** Statistics of up-regulated and down-regulated differentially expressed genes. **(C)** KEGG enrichment analysis of differentially expressed genes. The top 20 significantly enriched pathways are shown in the figure.

To screen the Psa resistance-related genes of kiwifruit, transcriptome sequencing was performed on the Psa extremely HR and HS plants screened from the R_1_F_2_ population. A total of 39.99 Gb Clean Data was obtained from six samples. The Clean Data of each sample reached 6.04 Gb, and the percentage of Q30 bases was above 90% (**Supplementary Table S1**). The Clean Reads of each sample were mapped to the reference genome *A. chinensis* Red 5. The mapping rates ranged from 74.39% to 83.00%; the uniquely mapped rate ranged from 71.62% to 79.77%. The sequence GC content was above 45% (**Supplementary Table S1**). These results indicated that the sequencing data of the six samples were of high quality and could be used for downstream analysis. Using false discovery rate (FDR) < 0.01 and Fold Change ≥ 2 as the screening criteria, a total of 581 DEGs were screened. Based on the functional annotation information, we annotated 563 DEGs, among which 403 were up-regulated and 160 were down-regulated (**Figures 2A, B**; **Supplementary Table S2**). Compared with HR plants, most of the DEGs in HS plants were up-regulated. To further analyze the function of DEGs, we performed a KEGG enrichment analysis. The top 20 enriched pathways contained 189 DEGs (**Figure 2C**; **Supplementary Table S3**). Among them, the highest enriched pathways were the plant hormone signal transduction (32 genes, 13.06%) and the plant-bacteria interaction pathway (31 genes, 12.65%) (**Figure 2C**; **Supplementary Table S3**). These results indicated that the genes related to plant hormone signal transduction might play important roles in the Psa resistance of kiwifruit.

### 3.3 Analysis of transcription factors in differentially expressed genes

**Figure 3:**
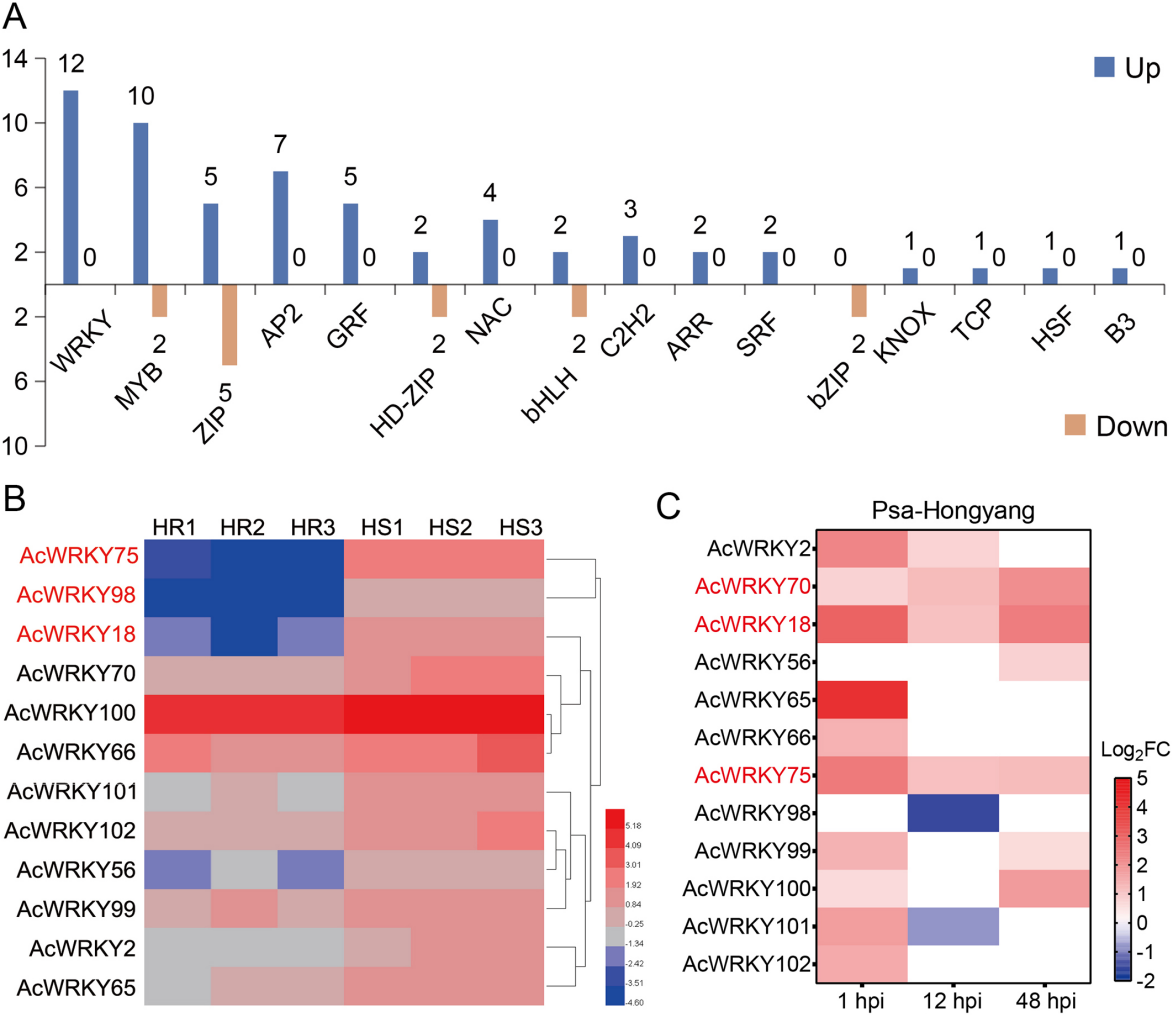
Statistics of differentially expressed transcription factors and expression heat maps of differentially expressed WRKY transcription factors. **(A)** Classification statistics of up-regulated and down-regulated transcription factors. **(B)** Expression heat map of WRKY genes in high-resistant and high-susceptible plants of R_1_F_2_ population. **(C)** Expression heat maps of WRKY transcription factors at 1, 12 and 48 hours post-inoculation (hpi) with Psa in ‘Hongyang’.

Gene classification of the DEGs showed that there were 71 TFs, among which the TF families such as WRKY (12 genes), MYB (12 genes) and ZIP (10 genes) were highly enriched (**Figure 3A**; **Supplementary Table S4**). It is worth noting that all the AcWRKY TFs were up-regulated in HS plants (**Figure 3A**), indicating that WRKY TFs may play an important role in Psa resistance of kiwifruit. Expression heat map analysis showed that *AcWRKY18*, *AcWRKY75* and *AcWRKY98* genes were significantly up-regulated (**Figure 3B**). ‘Hongyang’ is a highly susceptible variety of kiwifruit to Psa. Previously, we performed transcriptome analysis on ‘Hongyang’ at 0, 1, 12 and 48 hours post-inoculation (hpi) with Psa. The results showed that 10 of the 12 AcWRKY TFs were significantly up-regulated in 1 hpi, and the rest two displayed no significant differences in the expression levels. In 12 hpi, four AcWRKY TFs were up-regulated and 2 were down-regulated. In 48 hpi, six AcWRKY TFs were up-regulated, and the remaining six showed no obvious differences (**Figure 3C**). Among them, *AcWRKY70*, *AcWRKY18* and *AcWRKY75* were significantly up-regulated at 1, 12 and 48 hpi, and *AcWRKY18* and *AcWRKY75* showed the highest changes in the expression levels (**Figure 3C**). These results indicate that *AcWRKY18* and *AcWRKY75* genes may play an important regulatory role in the process of kiwifruit resistance to Psa.

### 3.4 Quantitative analysis of AcWRKY transcription factors

**Figure 4:**
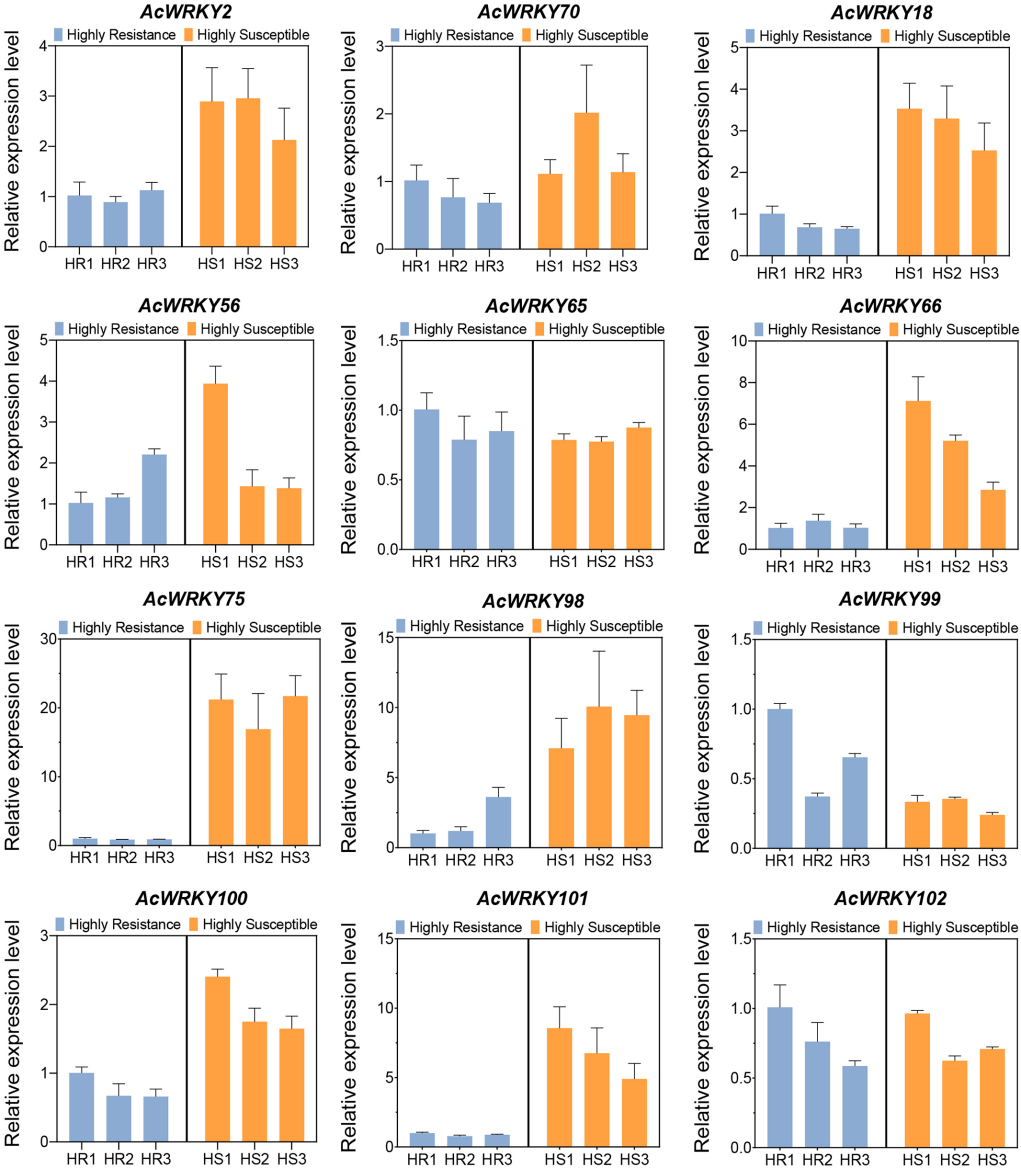
The qRT-PCR analysis of differentially expressed WRKY transcription factors in transcriptome sequencing. HR1/2/3 and HS1/2/3 were three highly resistant plant samples and three highly susceptible plant samples of the R_1_F_2_ population, respectively.

To further confirm the induced expression of AcWRKY TFs by Psa, we performed qRT-PCR analysis of 12 up-regulated WRKY TFs in 3 extremely HR and 3 extremely HS plants of the R_1_F_2_ population. The results showed that 10 of the 12 WRKY TFs were highly expressed in HS plants, which was consistent with the transcriptome results (**Figure 4**). Importantly, we found that *AcWRKY75* was most significantly up-regulated, and its expression level in HS plants was more than 20 times higher than that in HR plants (**Figure 4**). Therefore, it can be speculated that *AcWRKY75* is induced by Psa and plays an important role in the process of Psa infection.

### 3.5 Sequence polymorphism analysis of AcWRKY75 gene

**Figure 5:**
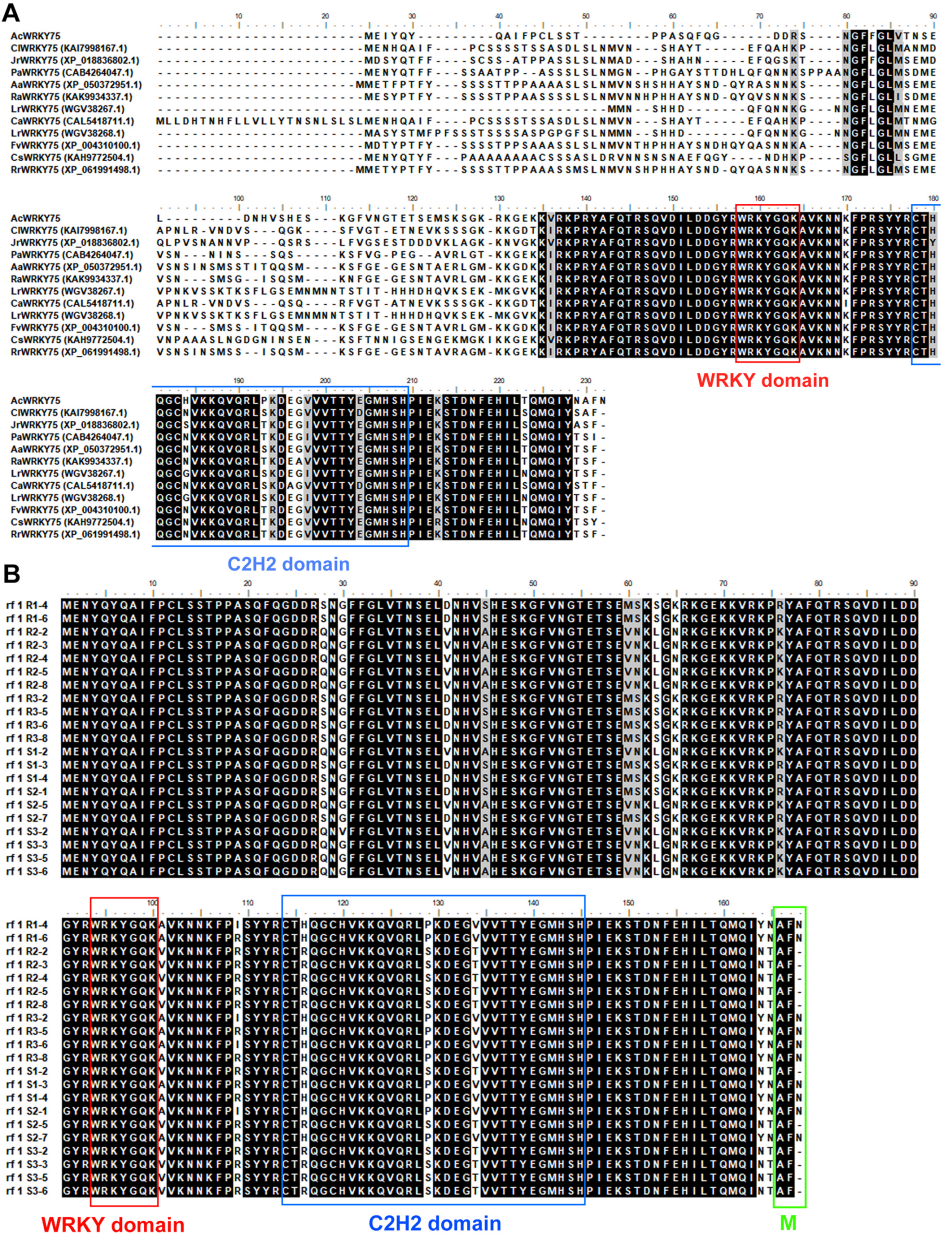
Sequence alignment of AcWRKY75 protein. **(A)** Multiple sequence alignment of AcWRKY75 protein and its homolog proteins. AcWRKY75, *Actinidiae chinesis*. ClWRKY75, *Camellia lanceoleosa*. JrWRKY75, *Juglans regia*. PaWRKY75, *Prunus armeniaca*. AaWRKY75, *Argentina anserina*. RaWRKY75, *Rubus argutus*. LrWRKY75, *Loropetalum chinense* var. *rubrum*. CaWRKY75, *Camellia sinensis*. FvWRKY75, *Fragaria vesca* subsp*. vesca*. CsWRKY75, *Citrus sinensis*. RrWRKY75, *Rosa rugosa*. **(B)** The protein sequence of AcWRKY75 from HR and HS plants of the R_1_F_2_ population. R1/2/3 are three extremely highly resistant plants, and S1/2/3 are three extremely highly susceptible plants. The conserved acid sequence WRKYGQK of the WRKY domain is highlighted in the red box; the zinc finger structure C2H2 is highlighted in the blue box; the single amino acid deletion site is highlighted in the green box.

To study the evolutionary characteristics of AcWRKY75, homologous phylogenetic trees were constructed in different species (**Supplementary Figure S3**). WRKY75 protein is conserved in different species of *Actinidia*, and is closely related to plants of *Juglans* and *Camellia* (**Supplementary Figure S3**). Multiple sequence alignment showed that AcWRKY75 is a group II WRKY transcription factor with a conserved WRKY domain and a zinc finger motif (C2H2) (**Figure 5A**). In addition, the C-terminus sequences of AcWRKY75 homologous protein are very conserved, while the N-terminal sequences harbor most of the variations (**Figure 5A**). Furthermore, to analyze the sequence polymorphism, we cloned and sequenced the genomic DNA of *AcWRKY75* gene in 3 HR plants and 3 HS plants of the R_1_F_2_ population. The results showed that the DNA sequence of *AcWRKY75* gene has obvious polymorphism, and there are many fragment deletions and single nucleotide mutations (**Supplementary Figure S4**). Most of these mutations are located in introns, and only one single base deletion is located in the second exon (**Supplementary Figure S4**), which causes a delay in translation termination and an extra amino acid (**Figure 5B**). However, these variations were present in both HR and HS, and no HR or HS-specific variations were found (**Figure 5B**). Whether these mutations affect the function of *AcWRKY75* gene remains to be further analyzed.

### 3.6 Subcellular localization and transcriptional activity analysis of AcWRKY75

**Figure 6:**
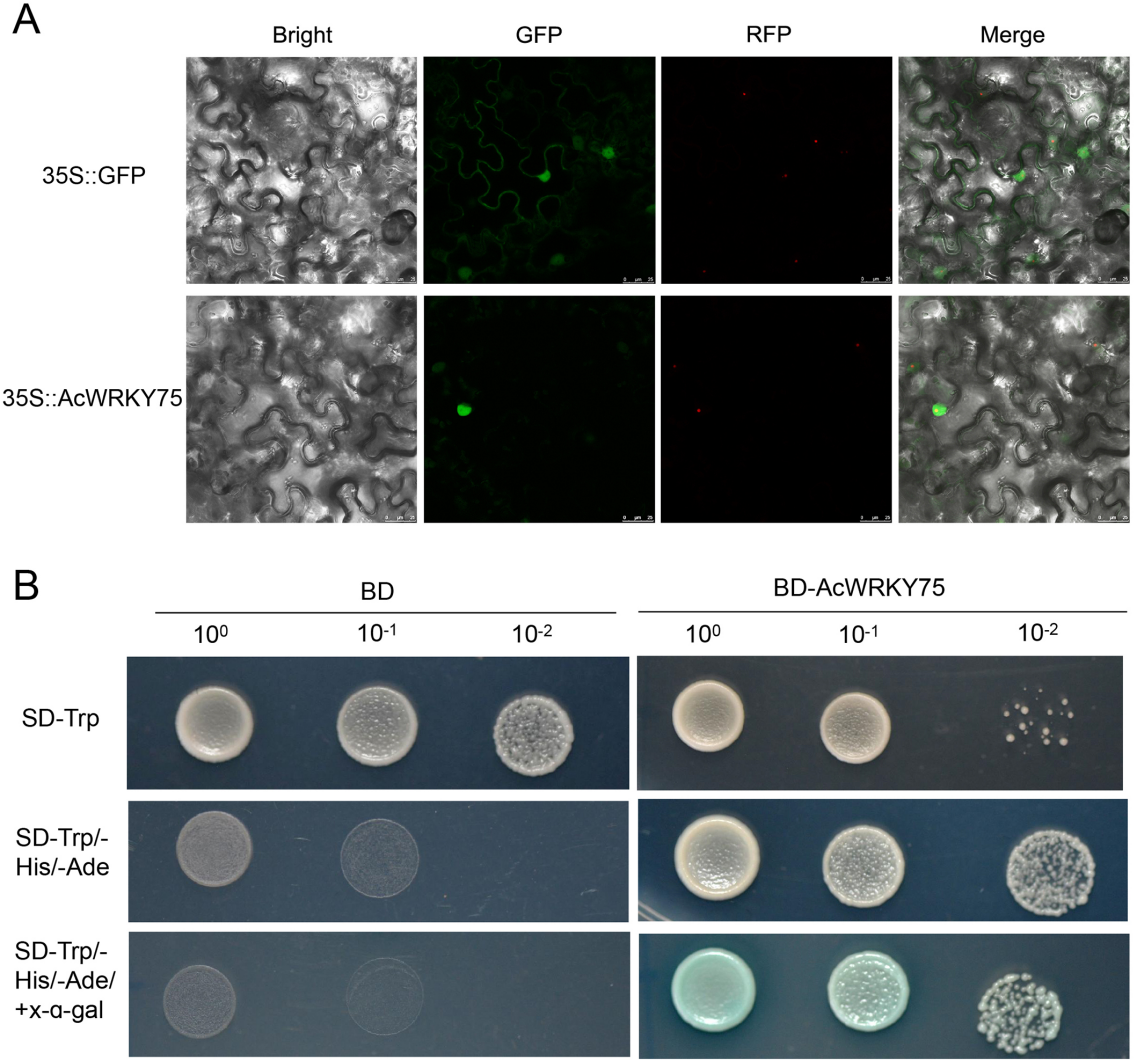
Subcellular localization and transcriptional activity analysis of AcWRKY75. **(A)** Subcellular localization of *AcWRKY75* in tobacco leaves. Green fluorescent protein (GFP) is fused to the C-terminal region of *AcWRKY75* gene, and the fusion protein is driven by the 35S promoter. Red fluorescent protein (RFP) label is used as a nuclear marker driven by 35S promoter, and 35S::GFP is used as positive control; **(B)** Analysis of AcWRKY75 transcriptional activity. Yeast cells transformed with BD‐AcWRKY75 grew well on yeast SD/−Trp/−His/-Ade plates and catalyzed X‐α‐gal into blue, indicating that AcWRKY75 has transcriptional activation activity. The BD empty vector is used as a negative control.

Transcriptome and qRT-PCR results have preliminarily confirmed that *AcWRKY75* gene was induced by Psa. To further explore the characteristics of *AcWRKY75* gene, we performed a subcellular localization analysis in *N. benthamiana*. The fluorescence signal showed that the AcWRKY75 protein was located in the nucleus (**Figure 6A**). In addition, we examined the transcriptional activity of *AcWRKY75* gene. The full-length CDS of the *AcWRKY75* gene was fused with the GAL4 DNA-binding domain, and the BD empty vector was used as a negative control. Only yeast cells harboring BD-AcWRKY75 grew well on the SD-Trp/-His/-Ade plate and catalyzed X-α-Gal into blue (**Figure 6B**). These results indicate that AcWRKY75 is a typical transcription factor with transcriptional activation activity. It may bind to the Psa resistance-related gene promoter to regulate its expression, thereby regulating the resistance of kiwifruit to Psa.

### 3.7 AcWRKY75 is a negative regulator of Psa resistance

**Figure 7:**
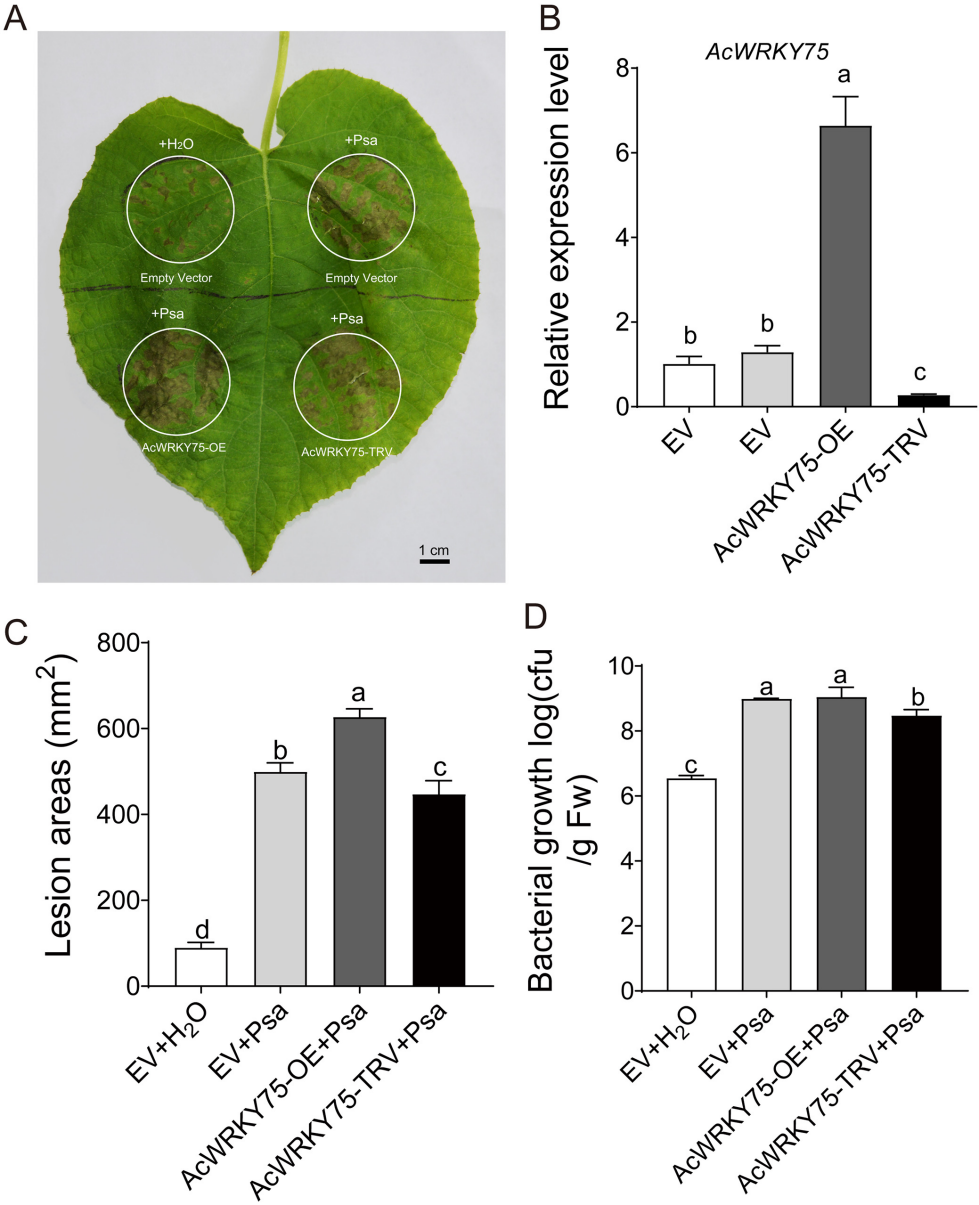
Transient overexpression and silencing of *AcWRKY75* in kiwifruit leaves. **(A)** *A. tumefaciens* infection and Psa inoculation. The *A. tumefaciens* solution was injected into the leaves in the white circle, and the empty vector was used as the control. The injection site was inoculated with Psa bacterial suspension 2 days after infection, and sterile water was used as a control. Two weeks after Psa inoculation, photos were taken and the lesion area was counted. Bars=1 cm. **(B)** Transcript levels of AcWRKY75 detected by qRT-PCR two days after infection. **(C)** Lesion areas in leaves after inoculation with Psa for Two weeks. ImageJ (V1.8.0) was used to calculate the lesion areas. **(D)** The biological content of bacteria Psa in leaves. Three independent biological replicates were used for each experiment. Different letters indicate statistical significance (P<0.05).

To better understand the role of *AcWRKY75* in Psa infection, we constructed overexpression and virus-induced gene silencing (VIGS) vectors, and then performed transient transformation in kiwifruit leaves, followed by Psa inoculation (**Figure 7A**). Two days post-transformation, we employed qRT-PCR to verify that the *AcWRKY75* gene was significantly up-regulated in the overexpression region and down-regulated in the VIGS region (**Figure 7B**). Two weeks after Psa infection, the overexpression of *AcWRKY75* resulted in a significant increase in the symptoms observed in kiwifruit leaves, whereas silencing *AcWRKY75* via VIGS led to a reduction in these symptoms (**Figures 7A, C**). Subsequently, we used the plate coating method to count the bacteria on the leaves of the control and different treatments. It found that *AcWRKY75* overexpressing leaves showed the highest amounts of bacteria, which was significantly higher than that of water control (**Figure 7D**). However, there were no significant differences between Psa-treated control leaves and *AcWRKY75* overexpressed leaves. It may be due to the number of Psa bacteria reaching the upper limit of growth. The amounts of bacteria in the leaves silently expressing the *AcWRKY75* gene were lower than that of the Psa-treated control (**Figure 7D**), which was consistent with the statistical analysis of the lesion areas of kiwifruit leaves. These results indicated that *AcWRKY75* can reduce the ability of kiwifruit leaves to resist Psa.

### 3.8 AcWRKY75 may regulate Psa resistance by regulating the expression of genes related to the hormone pathway

**Figure 8:**
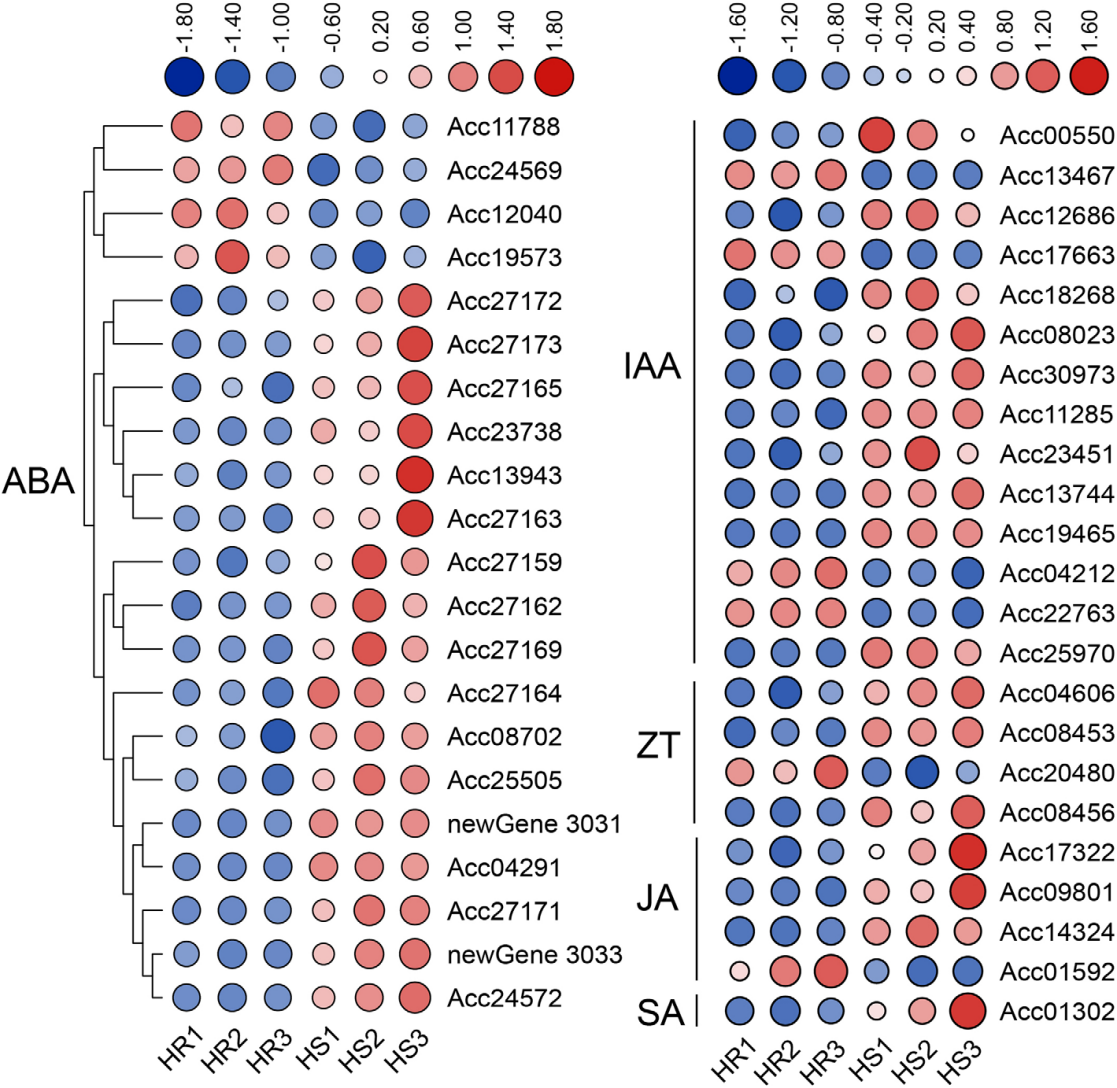
Heat map of hormone-related differentially expressed genes (DEGs) in the transcriptome. 21 DEGS were related to abscisic acid; 14 DEGS were related to auxin; 4 DEGS were related to zeatin; 4 DEGS were related to jasmonic acid; 1 DEG was related to salicylic acid.

**Figure 9:**
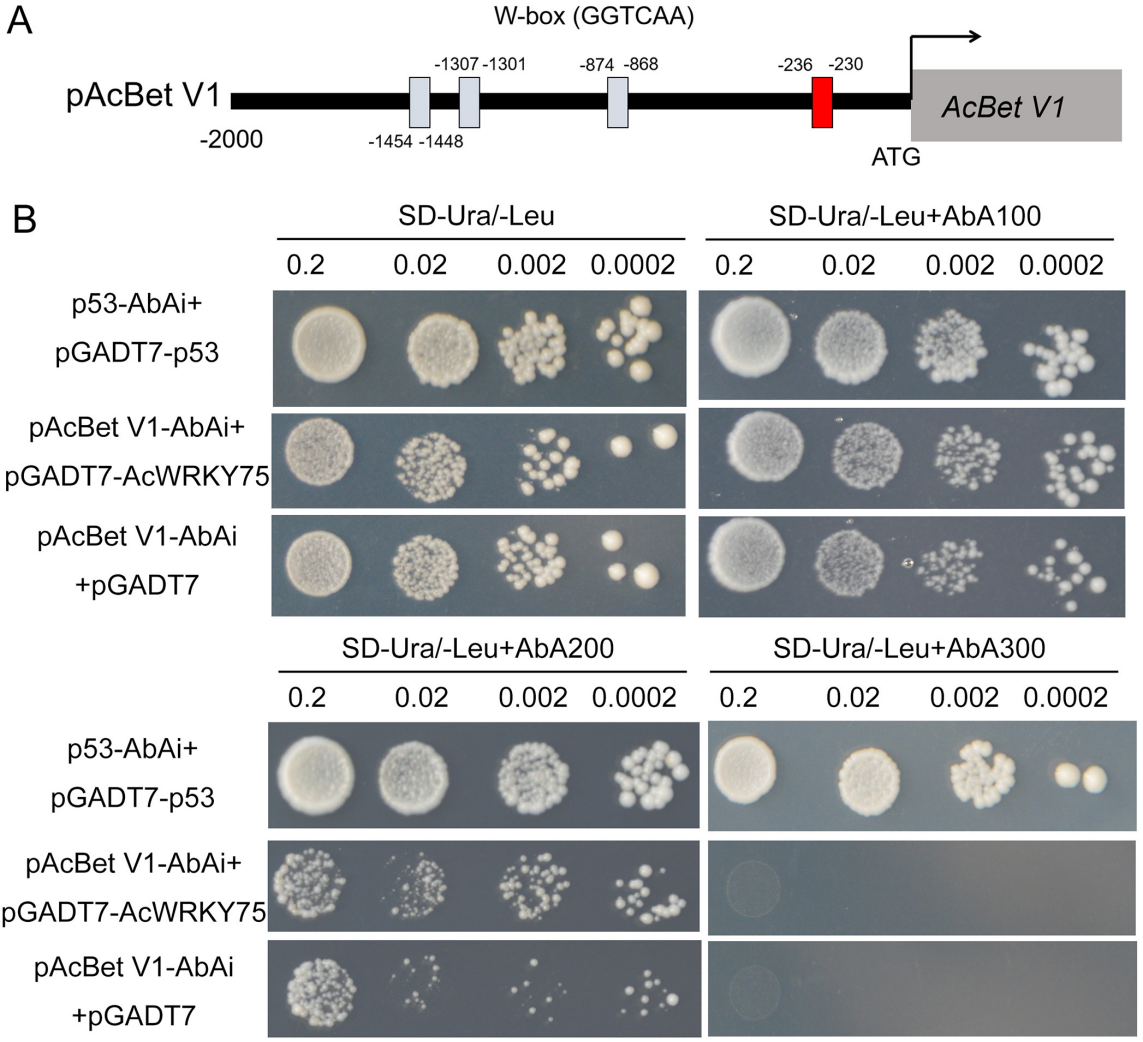
The interaction between the AcWRKY75 and *AcBet V1* promoter by yeast one-hybrid assay. **(A)** Four W-boxes of *AcBet V1* (Acc27163) gene promoter. The red box is the site for the yeast one-hybrid assay. **(B)** The yeast one-hybrid assay of AcWRKY75 and *AcBet V1* promoter. p53-AbAi+pGADT7-p53: positive control; pAcBet V1-AbAi+pGADT7: negative control. In SD-Ura/-Leu or SD-Ura/-Leu+AbA100 medium, the yeast of pAcBet V1-AbAi+pGADT7-AcWRKY75 grew well similar to positive control and negative control. In SD-Ura/-Leu+AbA200 medium, the yeast of pAcBet V1-AbAi+pGADT7-AcWRKY75 grew slightly stronger than negative, indicating a weak interaction between AcWRKY75 and *AcBet V1* promoter.

It has been reported that hormones are closely related to plant disease resistance (Pieterse et al., 2012). In this study, KEGG enrichment analysis found that DEGs were most significantly enriched in plant hormone signal transduction pathways (**Figure 2C**). Further analysis showed that the DEGs were mainly related to the metabolism of hormones such as auxin (14 genes), abscisic acid (21 genes), zeatin (4 genes), jasmonic acid (4 genes) and salicylic acid (1 gene) (**Supplementary Table S5**). Expression heat map showed that most genes were up-regulated in HS materials. For example, among the 21 ABA-related DEGs, only four were down-regulated and the rest were up-regulated; among the 14 auxin-related DEGs, 10 were up-regulated (**Figure 8**). It is worth noting that 14 ABA-related genes belong to the Pathogenesis-related protein Bet V1 family (**Supplementary Table S5**), indicating that the Bet V1 family proteins play an important role in the infection of Psa in kiwifruit. Promoter response element analysis showed that the Bet V1 family gene’s promoter contained multiple WRKY TF binding sites W-box, and the *AcBet V1* (Acc27163) gene promoter contained four W-box (**Figure 9A**). Furthermore, the *AcBet V1* gene was highly expressed in HS plants, similar to *AcWRKY75* (**Supplementary Table S2**). Therefore, we speculate that AcWRKY75 may bind to the *AcBet V1* gene promoter to regulate the Psa resistance of kiwifruit. Here, we cloned the W-box elements (-230 bp to -236 bp) closest to the start codon on the *AcBet V1* gene promoter and ligated into the pAbAi vector, and cloned the *AcWRKY75* coding region into the PGADT7 vector for yeast one-hybrid interaction verification. In SD-Ura/-Leu+AbA200 medium, the yeast of pAcBet V1-AbAi+pGADT7-AcWRKY75 grew slightly stronger than pGADT7 empty vector control, indicating that AcWRKY75 can weakly interact with the promoter of *AcBet V1* (**Figure 9B**). These results suggest that AcWRKY75 may bind to the promoter of ABA-related *AcBet V1* gene and promote its expression, thus regulating the Psa resistance in kiwifruit.

## 4 Discussion

The materials exhibiting varying resistance to canker disease serve as the foundation for screening disease-resistance genes. Song et al. analyzed the phenotype and transcriptome of resistant *A. eriantha* ‘huate’ and susceptible *A. chinensis* ‘Hongyang’ at 0, 12, 24, 48, 96 and 144 hours after inoculation with Psa (Song et al., 2019). Similarly, Qin et al. analyzed the transcriptome of the new shoots of the resistant cultivar ‘Maohua’ and susceptible cultivar ‘Hongyang’ after Psa infection (Qin et al., 2022). Zhao et al. screened 44 F_1_ hybrid lines derived from a cross between two *A. chinensis* lines and identified one resistant offspring RH12 and one susceptible offspring SH14 (Zhao et al., 2024). Most of the materials used in the reported studies are different resistant varieties or F_1_ populations of *A. chinensis* and *A. eriantha*. In this study, the six hybrid populations are BC_1_ and F_2_ populations of *A. deliciosa*. We used field investigation and *in vitro* branch resistance identification to evaluate the isolation of Psa resistance, and screened the resistant segregation population R_1_F_2_. The extremely HR and HS individuals were screened, and then transcriptome sequencing was performed. The materials used in this study are extremely HR and HS plants in the F_2_ population. Their Psa resistance is significantly different but the genetic background is similar, which is better than previous studies.

In recent years, some canker resistance-related genes have been identified For example, overexpression of *AcREM14*, *AcC3H1* and *AcWRKY94* enhance resistance to Psa by regulating the SA signaling pathways (Lu et al., 2024; Zhao et al., 2024). The TCP family genes *AcTCP2/9* were highly expressed in HR varieties, while *AcTCP6/12* were highly expressed in HS varieties (Li et al., 2024). Six LAC family genes, *AcLAC2/4/17/18/26/42*, were significantly induced after 5 days of inoculation with Psa (Zhong et al., 2023). Similarly, several TIFY family genes and *AeNPR1a* were also induced by Psa infection (Sun et al., 2020; Tao et al., 2022). On the contrary, some genes are negative regulators of canker resistance. For example, E3 ubiquitin ligase pub23 in kiwifruit interacts with trihelix TF GT1 and negatively regulates immune responses against Psa (Wang et al., 2024). *AcMYB16* acts as an inhibitory gene to regulate the biosynthesis of lignin in response to JA, thereby increasing the infection of kiwifruit leaves by Psa (Wang et al., 2021). In this study, we found a new negative regulator AcWRKY75, which may reduced the Psa resistance of kiwifruit through the hormone signaling pathway.

Multiple sequence alignment analysis showed that the *WRKY75* gene sequence was more conservative in different species, and all contained the WRKY domain and C2H2 domain. Functionally, the *WRKY75* gene is involved in a variety of biological processes, such as developmental processes, biotic and abiotic stresses. For example, in *Arabidopsis*, *WRKY75* is involved in regulating seed germination (Zhang et al., 2022a), leaf senescence (Guo et al., 2017; Zhang et al., 2022a), flowering (Zhang et al., 2018), root hair development (Devaiah et al., 2007; Rishmawi et al., 2014), phosphate acquisition (Devaiah and Raghothama, 2007), fungal pathogens *Botrytis cinerea* and *Alternaria brassicicola* disease resistance (Chen et al., 2021), Bacterial *Pseudomonas syringae* disease resistance (Encinas-Villarejo et al., 2009). In *Brassica napus* L, overexpression of *BnaA10.WRKY75* decreases cadmium and salt tolerance via increasing reactive oxygen species accumulation (Ping et al., 2024). In tomato, *WRKY75* maintains auxin homeostasis to promote defense against *Pseudomonas syringae* (Yang et al., 2024). In cassava, *MeWRKY75* and *MeWHYs* confer improved disease resistance against bacterial blight by forming an interacting complex (Liu et al., 2018b). In poplar, *WRKY75* regulates the development of adventitious roots, lateral buds and callus by modulating hydrogen peroxide content (Zhang et al., 2022b). In this study, AcWRKY75 is a group II WRKY TF, which was highly expressed in HS plants, and was significantly induced in ‘Hongyang’ after Psa inoculation. Transient overexpression of the *AcWRKY75* gene in kiwifruit leaves weakened the resistance to Psa, while silencing the *AcWRKY75* gene slightly enhanced the resistance. The role of *WRKY75* gene in kiwifruit canker disease was revealed for the first time in this study. Next, we will perform stable overexpression and gene knockout in kiwifruit to further verify the function of the *AcWRKY75* gene in negatively regulating canker disease resistance. The *AcWRKY75* gene has great application potential, and it may be possible to enhance the resistance of kiwifruit plants to Psa by knocking out this gene in the future. Hormones are closely related to plant disease resistance (Robert-Seilaniantz et al., 2011; Pieterse et al., 2012). In this study, transcriptome analysis found that DEGs were most significantly enriched in plant hormone signal transduction pathways. Studies has also shown that levels of many hormones, such as ethylene, jasmonic acid and salicylic acid in plants, will change after pathogen infection (Pieterse et al., 2012). It has been shown that JA accumulates in the sensitive line ‘Hongyang’, but decreases in the resistant line ‘Jinkui’ during Psa infection (Wang et al., 2021). The SA-controlled signaling pathway genes *NPR1*, *TGA*, and *PR1* displayed higher expression in the resistant line ‘Huate’ than in the sensitive ‘Hongyang’ (Song et al., 2019). Similarly, 4 JA-related genes among the DEGs were detected in this study. However, more DEGs are related to auxin and abscisic acid. We speculate that auxin and abscisic acid may also be related to the resistance of kiwifruit canker disease, but this needs further exploration. WRKY TFs can specifically recognize and bind to the DNA cis-acting element W-box (TTGACC/T) (Ciolkowski et al., 2008). Therefore, we analyzed the promoter sequences of hormone-related genes in DEGs and found that there were multiple W-box elements. The promoter of pathogenesis-related protein Bet V1 family gene *AcBet V1* (Acc27163) related to the ABA signaling pathway contained four W-boxes. This gene was highly expressed in Psa-susceptible varieties, similar to *AcWRKY75*. Yeast one-hybrid confirmed that AcWRKY75 has a weak interaction with the promoter of *AcBet V1*. Similarly, OsWRKY67 can directly activate rice pathogenesis-related protein PR gene to positively regulate rice blast resistance and bacterial blight resistance (Liu et al., 2018a). *JrWRKY21* can interact with JrPTI5L to promote the expression of pathogenesis-related protein *PR5L*, thereby improving the resistance of walnuts to anthracnose (Zhou et al., 2022). In the next step, we will continue to verify the interaction between AcWRKY75 and other W-box elements in the *AcBet V1* promoter or other genes to analyze the molecular mechanism of *AcWRKY75* regulating kiwifruit canker disease resistance.

## 5 Conclusion

In this study, we screened extremely HR and HS *A. deliciosa* plants from the resistant segregation population R_1_F_2_. The key candidate gene *AcWRKY75* related to Psa resistance was screened by comparative transcriptome and qRT-PCR analysis. It was highly expressed in HS plants and was significantly induced by Psa infection. Furthermore, AcWRKY75 is a group II WRKY transcription factor, which is localized in the nucleus and has transcriptional activation activity. Transient overexpression of *AcWRKY75* in kiwifruit leaves reduced Psa resistance, while silencing expression of *AcWRKY75* slightly enhanced Psa resistance. Additionally, AcWRKY75 had a weak interaction with the promoter of ABA-related DEG *AcBet v1* (Acc27163). Our study revealed that AcWRKY75 is a Psa-negative regulator, which may be involved in the regulation of kiwifruit canker disease by regulating the expression of hormone pathway-related genes.

## Data Availability

The transcriptome data of this study are deposited in the NCBI-SRA repository, accession number PRJNA1172983.

## Supplementary material

Supplementary Figure 1 Analysis of Psa resistance separation of six kiwifruit hybrid populations by *in vitro* identification. **(A–C, G–I)** Statistics of the length of the plant susceptible area. About 100 plants were identified in each population, and three replicates were identified for each plant. Three different color points represent three replicates, respectively. **(D–F, J–L)** The lesion of plant branches after inoculation with Psa.

Supplementary Figure 2 Statistics of disease resistance of six kiwifruit hybrid populations in the field. High resistance (HR): plants with no visible lesions on the leaves; medium resistance (M): plants with some lesions on the leaves; high sensitivity (HS): plants with lesions on almost all leaves.

Supplementary Figure 3 Phylogenetic analysis of AcWRKY75 protein in kiwifruit and its homologous proteins of other species.

Supplementary Figure 4 DNA sequence alignment of *AcWRKY75* gene in HR and HS plants of R_1_F_2_ population. R1/2/3 are three extremely high-resistant plants, and S1/2/3 are three extremely high-susceptible plants. In the red box are the exons of the *AcWRKY75* gene.
