# Supplementary figures and images for: Transcriptome analysis revealed that AcWRKY75 transcription factor reduced the resistance of kiwifruit to Pseudomonas syringae pv. actinidiae
Source: Front Plant Sci. 2024 Oct 24;15:1488572. doi: 10.3389/fpls.2024.1488572 (PMC11540699; doi:10.3389/fpls.2024.1488572)

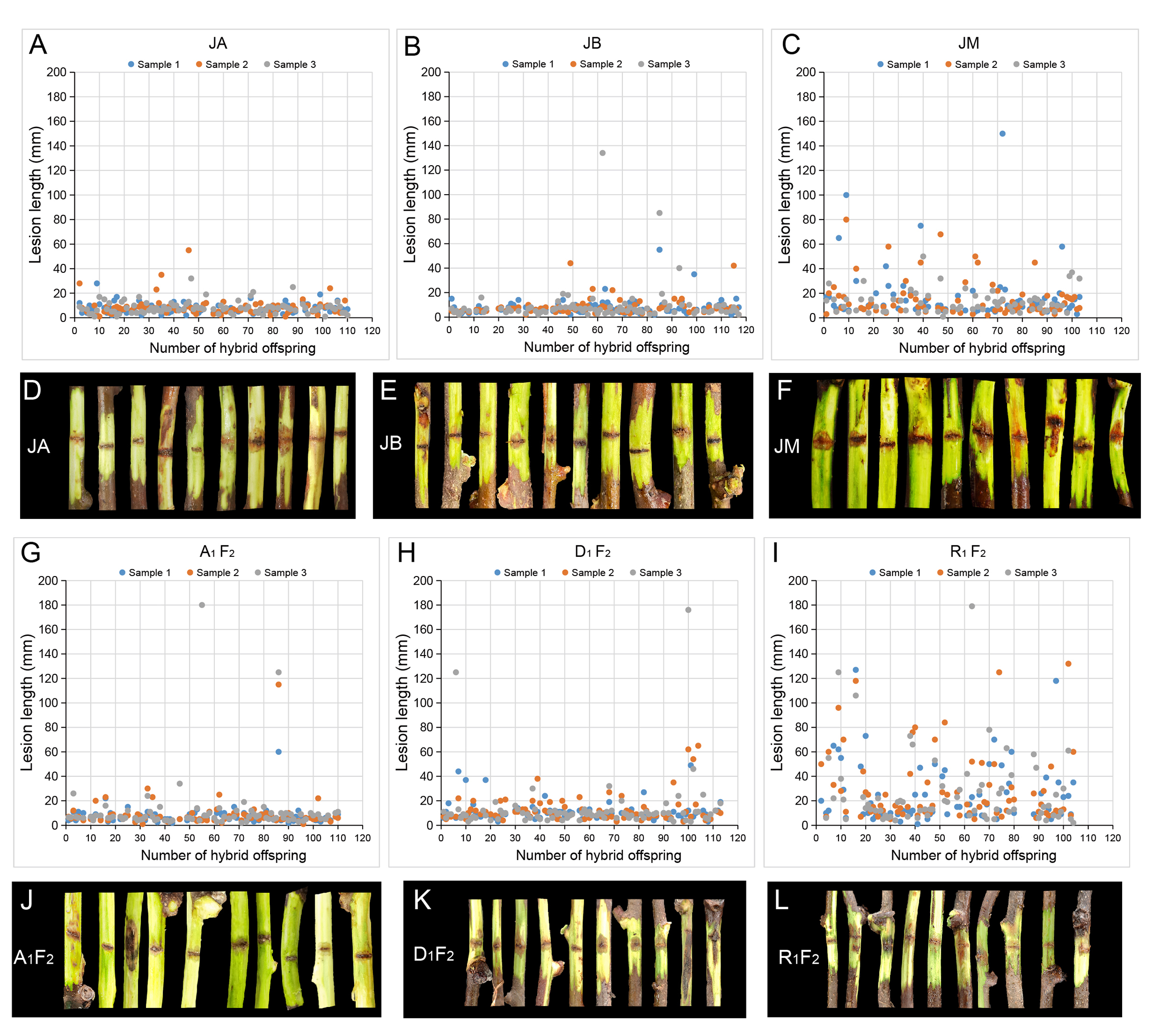

Supplement: Supplementary Figure 1 — Analysis of Psa resistance separation of six kiwifruit hybrid populations by in vitro identification. (A–C, G–I) Statistics of the length of the plant susceptible area. About 100 plants were identified in each population, and three replicates were identified for each plant. Three different color points represent three replicates, respectively. (D–F, J–L) The lesion of plant branches after inoculation with Psa. [file Image1.jpeg]

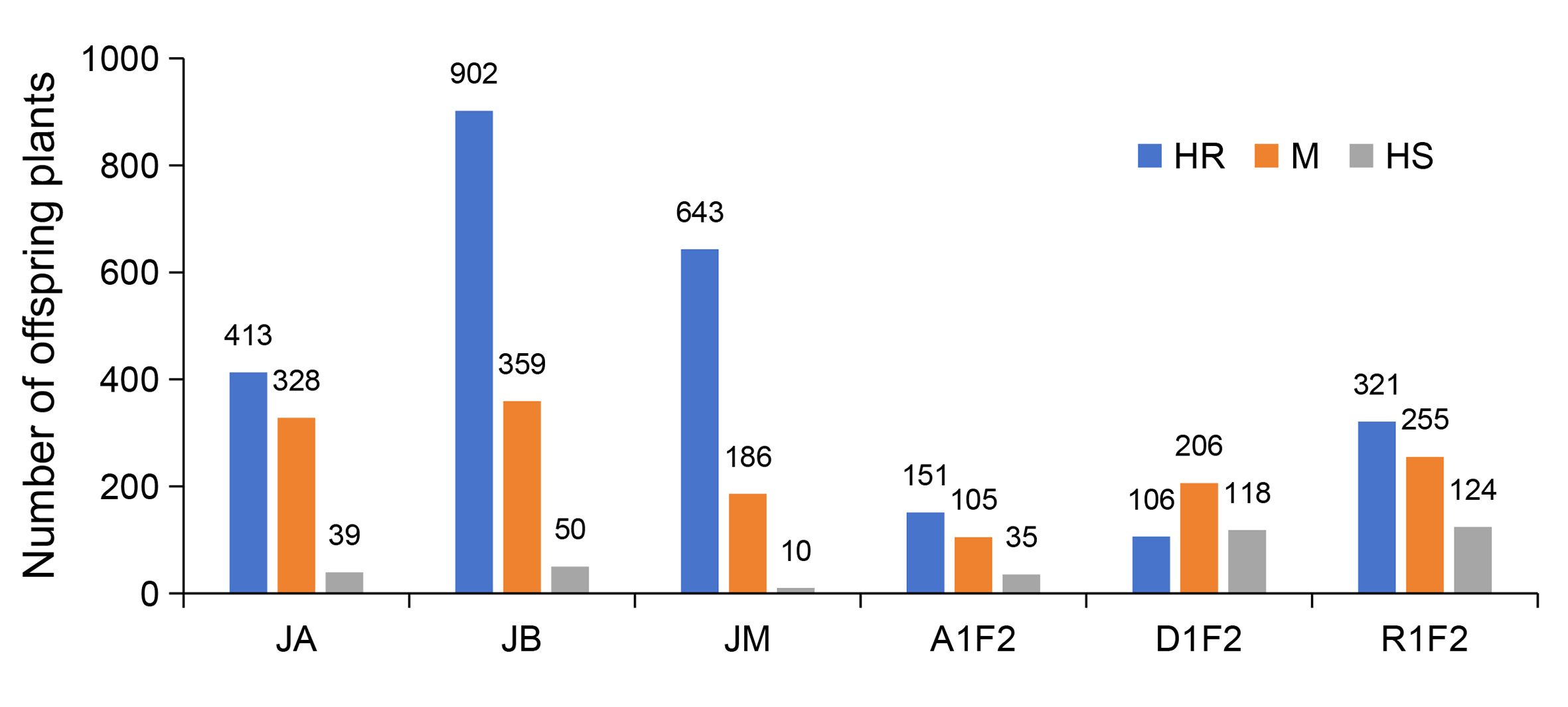

Supplement: Supplementary Figure 2 — Statistics of disease resistance of six kiwifruit hybrid populations in the field. High resistance (HR): plants with no visible lesions on the leaves; medium resistance (M): plants with some lesions on the leaves; high sensitivity (HS): plants with lesions on almost all leaves. [file Image2.jpeg]

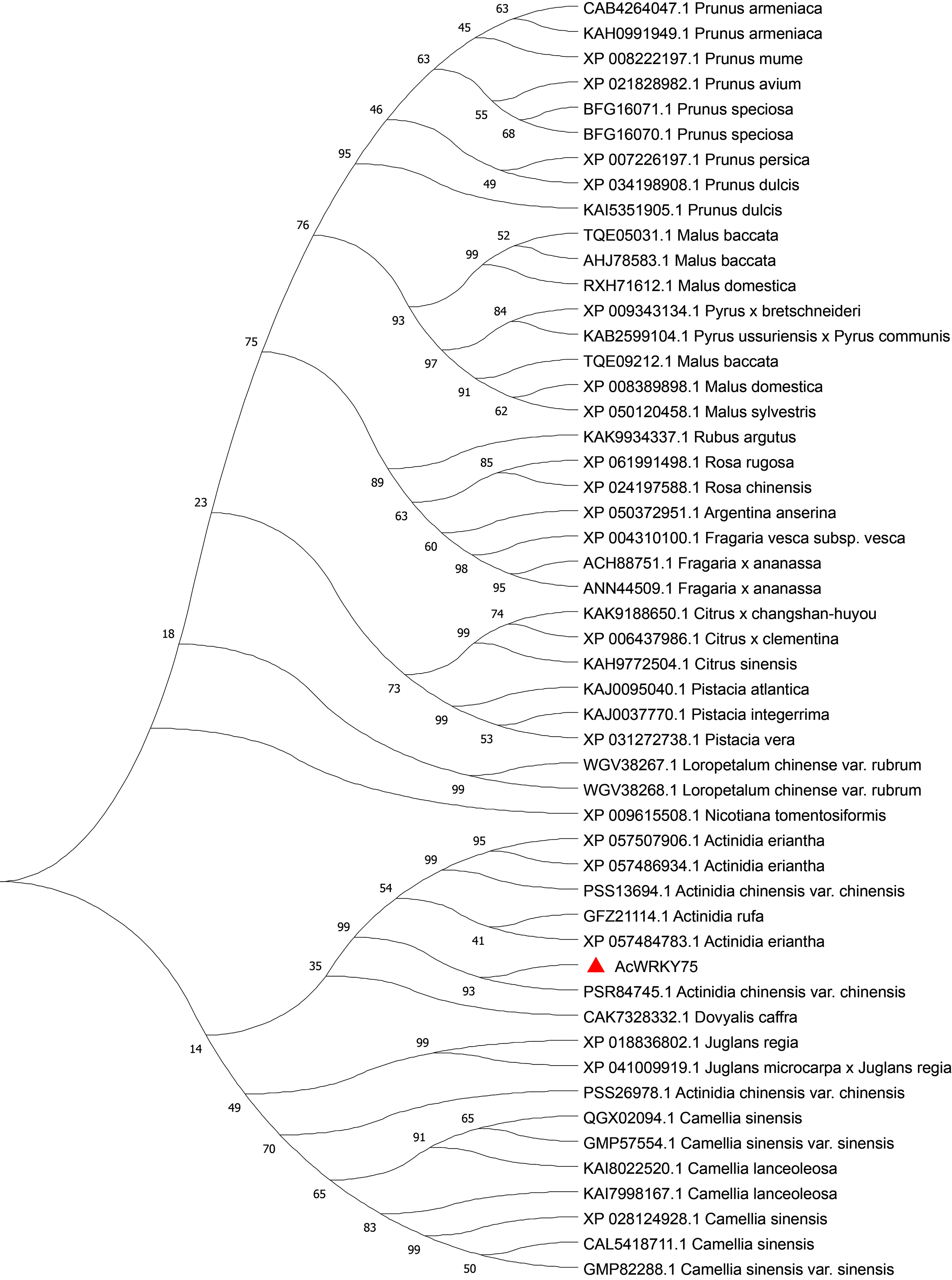

Supplement: Supplementary Figure 3 — Phylogenetic analysis of AcWRKY75 protein in kiwifruit and its homologous proteins of other species. [file Image3.jpeg]

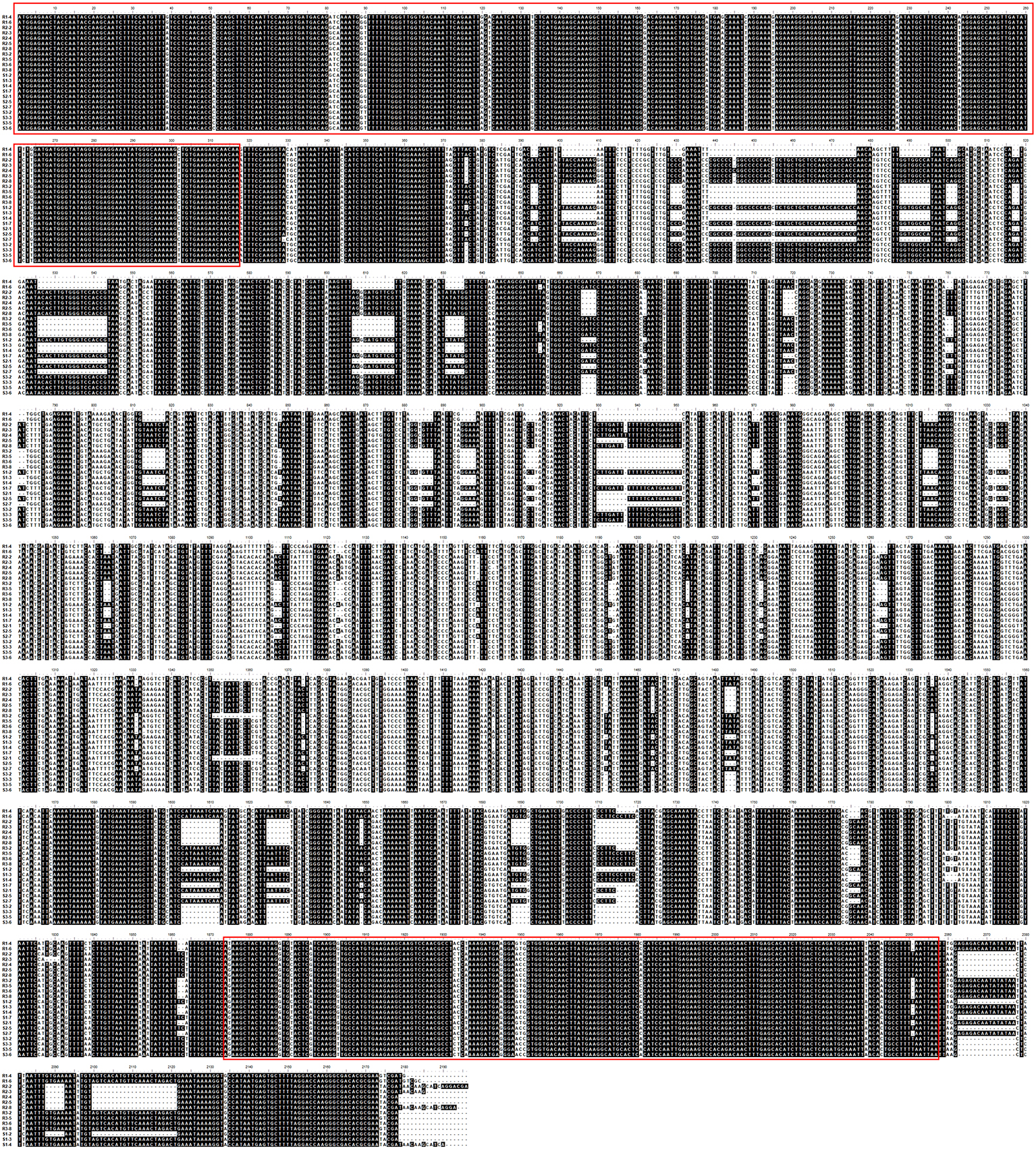

Supplement: Supplementary Figure 4 — DNA sequence alignment of AcWRKY75 gene in HR and HS plants of R1F2 population. R1/2/3 are three extremely high-resistant plants, and S1/2/3 are three extremely high-susceptible plants. In the red box are the exons of the AcWRKY75 gene. [file Image4.jpeg]
